# Imagined Speech Brain–Computer Interface: A Task-Oriented Review of Neural Decoding

**DOI:** 10.3390/s26103212

**Published:** 2026-05-19

**Authors:** Haodong Zhang, Wai Ting Siok, Nizhuan Wang

**Affiliations:** Department of Language Science and Technology, The Hong Kong Polytechnic University, Hung Hom, Kowloon, Hong Kong SAR, China; hawdong.zhang@polyu.edu.hk

**Keywords:** imagined speech, brain–computer interface (BCI), neural decoding, task-oriented review, output pathway, closed-set, open-vocabulary

## Abstract

Imagined speech decoding has attracted growing interest in brain–computer interface (BCI) research, as it may enable language-related information to be recovered from non-overt neural activity. Current studies in this area are often treated as a single, unified research problem, despite substantial differences in decoding target, output constraints, and system output forms. This review examines recent imagined speech decoding research from a task-oriented perspective, with a focus on how different neural decoding tasks are defined, constrained by their output spaces, and expressed through different output pathways. The included studies are organized into four main task levels: semantic/intent, phoneme/syllable, word, and sentence/language decoding. They are further compared along two auxiliary dimensions: output-space property and output pathway, with particular attention to closed-set and open-vocabulary settings. The review shows that current studies span markedly different linguistic granularities and communication objectives, from low-bandwidth intent recognition to text or speech reconstruction. Finally, it concludes that imagined speech should not be treated as a single homogeneous decoding problem, and that a task-oriented framework provides a clearer basis for comparing heterogeneous studies and guiding future communication-oriented BCI research.

## 1. Introduction

Brain–computer interfaces (BCIs) aim to establish direct communication pathways between neural activity and external devices, offering a promising route for restoring interaction in individuals with severe motor or speech impairments [1,2,3]. Among different BCI paradigms, speech-related neural decoding has attracted increasing attention because it moves beyond selection-based interfaces and toward more natural, flexible, and language-driven communication. In this context, imagined speech, namely the internal generation of speech without overt articulation or acoustic output, has emerged as an important research direction for brain–computer communication [2,4,5].

Beyond stimulus-driven spelling systems such as P300- or SSVEP-based interfaces [6,7], motor imagery BCI represents a widely studied internally generated control paradigm. Motor imagery and imagined speech share a broad conceptual similarity as internally generated BCI paradigms, because both rely on imagined mental processes rather than overt physical execution. However, motor imagery typically involves the imagination of movement and is often used for control-oriented applications, such as brain-controlled vehicles and rehabilitation robot control [8,9], whereas imagined speech involves the internal generation of language-related content and holds the potential to support more intuitive and expressive communication. This makes imagined speech particularly relevant for users who retain language intention but have lost the ability to produce intelligible speech [2,4,5,10]. However, imagined speech decoding remains substantially more challenging than overt speech decoding. The absence of clear behavioral output, weak and variable neural signatures, subject-specific differences, and the lack of stable temporal alignment all contribute to the difficulty of building reliable and generalizable systems [2,3,4,10,11,12,13].

Existing reviews offer valuable overviews of this field, addressing neural signal acquisition, preprocessing, feature extraction, datasets, and decoding models [2,3,4,5,10,11,12,13,14]. While some reviews have acknowledged that imagined speech studies target various linguistic units, from vowels and phonemes to words and sentences [4,5,10,12,14], such distinctions are usually mentioned as part of broader process-oriented summaries (which are important for understanding how imagined speech systems are built) rather than being developed into a unified task-oriented framework for literature organization and boundary clarification [2,4,5,12]. Nevertheless, a key difficulty in the literature is that the studies being compared often do not aim to decode the same type of linguistic target. Some focus on semantic intent or communicative categories, others target phonemes or syllables, many operate at the word level, and more recent work has begun exploring sentence-level reconstruction, text generation, and speech synthesis [4,10,12,14,15].

This heterogeneity has several consequences. First, results from different studies are often compared without adequate consideration of task-level differences. Second, conceptually distinct settings, such as fixed-sentence classification versus open-vocabulary language reconstruction, are sometimes conflated. Third, output pathways, including neural-signal-to-label, neural-signal-to-text, neural-signal-to-speech, and cascaded neural-signal→text→speech systems, are frequently discussed together without clearly distinguishing their underlying goals and assumptions. As a result, imagined speech is often summarized as a relatively unified research direction, even though the underlying studies differ substantially in terms of decoding target level, output constraints, and communication objective across studies [2,4,5,12,14]. These issues make it difficult to assess real progress in imagined speech research and to identify which directions are most meaningful for practical brain–computer communication.

To address this problem, the present review adopts a task-oriented framework for organizing the imagined speech literature. Instead of focusing solely on the methodological pipeline, the review first asks what is being decoded. Based on the linguistic level of the decoding target, existing studies are organized into four main categories: semantic or intent-level, phoneme or syllable-level, word-level, and sentence or language-level decoding. To further improve comparability, two auxiliary dimensions are introduced. The first is the output-space property, which distinguishes closed-set from open-vocabulary settings. The second is the output pathway, which distinguishes neural-signal-to-label, neural-signal-to-text, neural-signal-to-speech, and cascaded neural-signal→text→speech systems. This framework is intended not as a rigid universal taxonomy, but rather as a practical structure to clarify task boundaries, enable more consistent interpretation of results, and identify underexplored directions in the field.

The central argument of this review is that imagined speech should not be treated as a single decoding problem, but instead as a family of related yet distinct tasks that differ in linguistic granularity, output constraints, and system objectives [16]. From this perspective, questions such as whether a study decodes words or intentions, predicts labels or reconstructs speech, and operates in a fixed or open output space are not secondary implementation details. They fundamentally shape the difficulty, interpretation, and communicative significance of the task.

To clarify the positioning of the present review relative to existing literature, Table 1 summarizes representative existing reviews related to imagined speech decoding and closely related speech-BCI topics. These representative reviews were selected to illustrate the major ways in which prior reviews have organized this field, including methodological, dataset-oriented, reconstruction-oriented, classification-oriented, and protocol-oriented perspectives. In contrast, the present review adopts task level, output-space property, and output pathway as the primary organizing dimensions, thereby focusing on what is being decoded, how constrained the output space is, and how the decoded content is expressed.

Accordingly, this review makes the following contributions. First, unlike existing reviews that mainly summarize imagined speech decoding from methodological, dataset-oriented, classification-oriented, reconstruction-oriented, or protocol-oriented perspectives, this review reorganizes the literature around a task-oriented framework centered on what is being decoded. Second, it distinguishes four task levels, namely semantic/intent-level, phoneme/syllable-level, word-level, and sentence/language-level decoding, and further interprets them through two auxiliary dimensions: output-space property and output pathway. Third, it clarifies boundary cases that are frequently conflated in existing discussions, including semantic intent versus lexical decoding, sentence-level tasks versus open-vocabulary settings, and output pathways versus primary task categories. Finally, it relates these tasks and output distinctions to practical brain–computer communication, highlighting the trade-offs among expressiveness, robustness, transparency, and communicative usefulness.

In the present review, imagined speech is used as the primary organizing term for studies on language-related decoding from non-overt neural activity, while closely related studies are included when relevant to the review scope. Because terminology in this area is not fully consistent across studies, related terms such as inner speech, covert speech, speech imagery, and silent speech are sometimes used to describe overlapping but not identical paradigms. Therefore, inclusion was guided primarily by decoding the objective and experimental setting rather than by terminology alone. To ensure sufficient coverage, the reviewed literature was collected from major academic databases and screened according to predefined inclusion and exclusion criteria. The detailed scope definition, search strategy, screening procedure, and Preferred Reporting Items for Systematic Reviews and Meta-Analyses (PRISMA) 2020-based study selection process are presented in Section 2.

The remainder of this review is organized as follows. Section 2 clarifies the scope of the review and the basic challenges of imagined speech research. Section 3 introduces the task-oriented framework adopted in this review. Section 4 discusses the two auxiliary dimensions and provides cross-category analysis. Section 5 presents the overall discussion on major methodological and application-oriented issues. Finally, Section 6 concludes the review.

## 2. Literature Search, Selection Strategy, and Basic Background

### 2.1. Literature Search Strategy

**Figure 1:**
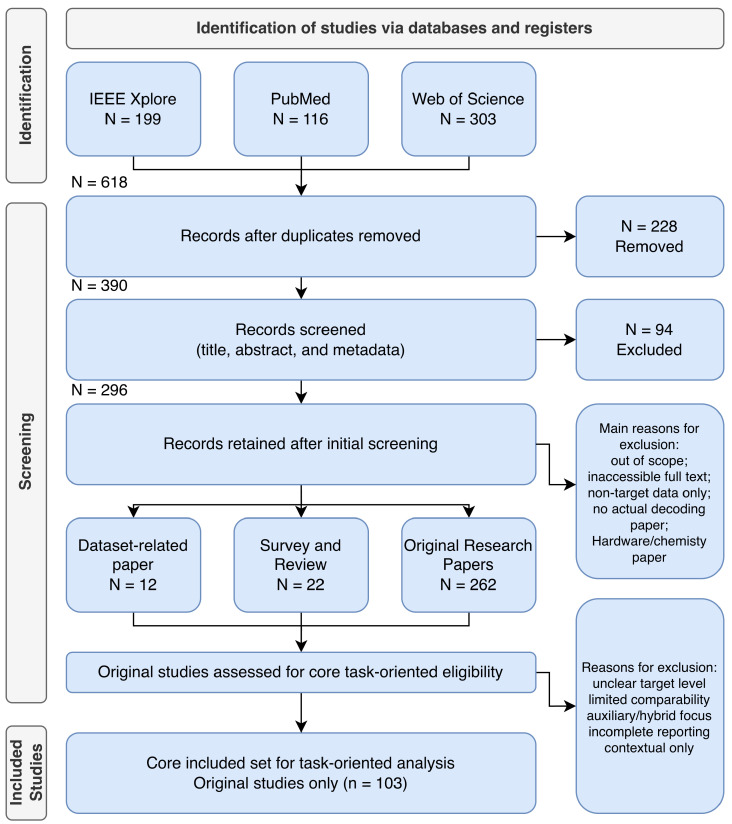
PRISMA flow diagram of the study selection process for the present review.

This review uses the Preferred Reporting Items for Systematic Reviews and Meta-Analyses (PRISMA) 2020 reporting logic to present the literature identification and screening process for imagined speech decoding studies [23,24]. In this review, PRISMA 2020 is used primarily as a reporting structure for documenting record identification, screening, and final inclusion, while the main analytical focus lies in the task-oriented organization and interpretation of the recent core evidence pool. The overall study selection process is summarized in Figure 1. Considering the rapid development of this area in recent years, especially with advances in deep learning, multimodal modeling, and foundation-model- or large language model (LLM)-related approaches, the primary search window for constructing the core evidence pool was set from 1 January 2020 to 6 February 2026. We acknowledge that important imagined speech and related speech-BCI studies were published before 2020; therefore, pre-2020 studies with clear foundational significance in task definition, experimental paradigm, dataset construction, or methodological development were retained as background references where necessary. However, these earlier studies were not counted in the main screening statistics, which were used to define the recent core evidence pool analyzed in this review.

The core database search covered Web of Science Core Collection, PubMed, and IEEE Xplore. Google Scholar was used only for supplementary retrieval and cross-checking and was not included in the main study selection counting process, organized according to PRISMA 2020 reporting logic. Search fields primarily included title, abstract, and keywords. A unified search strategy was constructed by combining three blocks of terms, namely task-related terms, signal-modality terms, and decoding-method terms. In general form, the search logic can be summarized as follows: imagined speech-related terms, including imagined speech, inner speech, speech imagery, covert speech, and silent speech, were combined with modality-related terms, including electroencephalography (EEG), electrocorticography (ECoG), stereoelectroencephalography (sEEG), magnetoencephalography (MEG), functional magnetic resonance imaging (fMRI), functional near-infrared spectroscopy (fNIRS), and brain–computer interface (BCI), and with method-related terms such as decoding, classification, recognition, neural decoding, machine learning, deep learning, CNN, RNN, LSTM, transformer, transfer learning, and domain adaptation. The exact syntax was adapted to the search rules of each database while keeping the core search logic consistent. The same three-block search logic was applied across databases, but the executable syntax was adapted to each database’s field structure and query rules. To improve reproducibility, the full database-specific search strings, search fields, publication windows, document-type restrictions, language restrictions, and query dates used in the main retrieval stage are provided in Appendix Table A1, Table A2 and Table A3.

Although imagined speech is used as the primary organizing term in the present review, the search strategy was intentionally broader than a single-term query. This broader retrieval strategy was adopted because a number of relevant studies use partially overlapping terminology while still addressing closely related non-overt speech decoding problems. However, the final inclusion decision was guided primarily by the actual experimental objective and decoding target of each study rather than by terminology alone.

### 2.2. Screening Procedure, Inclusion/Exclusion Criteria, and Evidence Organization

In the main search stage, a total of 618 records were identified, including 199 from IEEE Xplore, 116 from PubMed, and 303 from Web of Science. All records were imported into Zotero for management, and duplicate removal was performed through a combination of automatic merging and manual verification. After deduplication, 390 unique records remained, indicating that 228 duplicate records were removed.

The 390 remaining records were then screened based on title, abstract, and basic metadata, leading to the exclusion of 94 records at the initial screening stage. The main reasons for exclusion included inaccessible full text, topics not directly related to imagined, covert, inner, or silent speech decoding, studies focusing only on non-target signals such as electromyography (EMG) without neural decoding, and papers without actual decoding experiments, such as hardware, materials, or acquisition-device studies.

After the first-round screening, 296 records were retained as the initial evidence pool for the review. These were then separated by study type, including 12 dataset-related papers, 22 review-type records, and 262 original research papers. Because the present review focuses on core task-oriented imagined speech decoding studies, the original research papers were further assessed for task-level relevance and comparability. Studies with unclear target level, limited comparability, auxiliary or hybrid task focus, incomplete reporting, or contextual relevance only were excluded at this stage. As a result, 103 original studies were retained as the final core set for task-oriented analysis. Dataset and review papers were used separately for resource analysis and background synthesis rather than being merged into the core task-oriented methodological analysis. The study selection process followed PRISMA 2020 reporting logic and is summarized in a customized flow diagram.

The main inclusion criteria were as follows. First, the study had to be directly related to neural decoding of imagined speech, inner speech, covert speech, or silent speech. Second, the study had to be a peer-reviewed journal paper or conference paper. Third, it had to report a clearly defined task, methodological pipeline, and quantitative results. Fourth, the full text had to be accessible.

Studies were excluded if they were not directly related to imagined or non-overt speech decoding, including studies focusing only on overt speech, only on motor imagery, or only on general language psychology without a decoding objective. Non-formal academic materials such as abstracts, posters, tutorials, patents, and news reports were excluded. Duplicated or redundant publications were excluded as well. Studies lacking sufficient experimental detail or key quantitative results for meaningful comparison were also excluded. In addition, papers focusing only on materials, hardware, or system construction without actual decoding experiments were removed from the core evidence pool. To reduce discretionary interpretation, the final exclusion categories were applied according to the primary decoding objective and the information needed for task-oriented comparison. Specifically, studies were excluded from the core evidence pool when the decoded target could not be assigned to the proposed task levels, when imagined speech decoding was not the primary experimental objective, or when the task setting, label space, evaluation protocol, or quantitative results were insufficient for meaningful cross-study interpretation.

For each included study, a standardized extraction template was used to record bibliographic information, task granularity and label space, acquisition modality and experimental setting, feature extraction and model architecture, evaluation protocol, performance metrics, and reproducibility-related details. Particular attention was given to whether the reported results were based on within-subject, cross-session, or cross-subject evaluation, and whether the data split strategy and leakage-control procedure were clearly described. These extracted items were used to support the assignment of each study to the task-level, output-space, and output-pathway categories used in the present review. For studies with mixed targets or multiple output forms, classification was based on the dominant decoding objective reported in the original study, while secondary characteristics were retained for interpretation.

To avoid simple comparison based only on headline accuracy, the extracted evidence was also organized with attention to methodological rigor. Studies with cross-subject or cross-session evaluation and clearly described split strategies were treated as stronger evidence for generalization. Studies with complete within-subject evaluation and sufficiently reproducible methodological reporting were treated as useful but more limited evidence. Studies with incomplete reporting or only very limited quantitative results were used mainly for trend-level reference rather than strong comparative claims. This evidence interpretation was used to avoid treating small-sample, closed-set, or subject-dependent results as directly comparable indicators of generalizable imagined speech decoding performance.

The extracted studies were later mapped onto the main technical pathways discussed in this review, namely neural-signal-to-label, neural-signal-to-text, and neural-signal-to-speech, including cascaded neural-signal→text→speech systems. This organization helps maintain a consistent basis for later comparison across task definitions, evaluation settings, and communication-oriented system goals. In this way, the literature search and extraction process was directly linked to the task-oriented framework, ensuring that the final evidence pool was not only collected systematically but also organized according to comparable task definitions, output-space properties, and output pathways.

### 2.3. Basic Challenges of Imagined Speech Decoding

**Figure 2:**
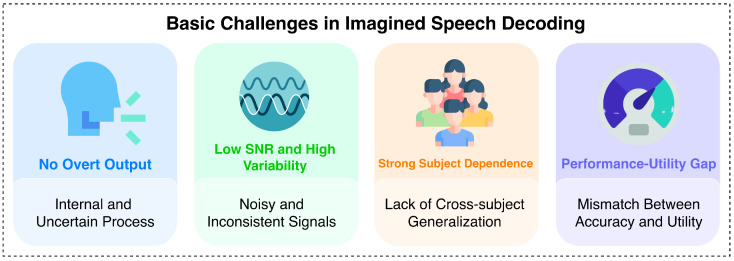
Overview of the basic challenges in imagined speech decoding. The figure summarizes the major difficulties that shape current imagined speech research and affect the robustness, evaluation, and practical usefulness of brain–computer communication systems.

Imagined speech decoding remains substantially more difficult than many conventional BCI paradigms [2,3,4,5,11,12,13]. An overview of the main challenges is presented in Figure 2. The first challenge is the absence of overt behavioral output. In tasks such as motor execution or overt speech, observable behavior provides a relatively clear anchor for segmentation, timing, and label verification. In imagined speech, however, the intended linguistic event unfolds internally, which makes temporal alignment much less certain and weakens the reliability of trial-level supervision.

A second challenge is the low signal-to-noise ratio and high variability of non-invasive neural recordings [2,4,11,12,13]. EEG-based imagined speech signals are weak, susceptible to noise and artifacts, and often highly variable across trials, sessions, and subjects. This makes it difficult to determine whether observed decoding performance reflects robust speech-related representation or merely limited task-specific separability under tightly controlled settings. The problem becomes more severe when studies move from small closed-set tasks toward higher-level language reconstruction.

A third challenge is substantial subject dependence, which leads to limited cross-subject generalization [2,3,11,12,13]. Neural correlates of internally generated language vary across individuals, and as a result, models trained on one subject often generalize poorly to others. Accordingly, much of the literature remains focused on within-subject decoding, an approach that is useful for feasibility testing but often offers limited insights into how well such systems would perform in real-world communication applications.

A fourth challenge concerns the gap between task-constrained performance and real-world communicative usefulness. A system may achieve relatively high accuracy in a small closed-set experiment yet offer limited practical expressiveness. Conversely, systems aiming at richer outputs, such as text or speech reconstruction, are often more attractive from a communication perspective but harder to validate, as their outputs can be influenced by language priors, auxiliary signals, or post-processing modules. These factors make imagined speech research both methodologically demanding and conceptually difficult to compare across studies.

### 2.4. Why a Task-Oriented Framework Is Needed

The above challenges are compounded by the fact that imagined speech is often discussed as if it were a unified decoding problem, even though the underlying studies differ substantially in terms of target level, output constraints, and system objective. Existing reviews have highlighted differences in acquisition modality, preprocessing, feature engineering, and classifier design [2,3,4,5,10,11,12,13,14]. However, comparing studies that decode semantic intent, phonological units, single words, and sentence-level content without clearly distinguishing task levels can lead to misleading conclusions.

For this reason, a task-oriented framework is needed not only to organize the literature more clearly, but also to make future comparisons more meaningful. Such a framework helps distinguish what linguistic content is being decoded, whether the task is constrained or open-ended, and how the decoded result is ultimately expressed. These questions are central to understanding both methodological difficulty and communication relevance. The following sections, therefore, adopt a task-oriented structure in which the main organizing axis is the linguistic level of the decoding target, while output-space property and output pathway are treated as auxiliary dimensions for cross-study interpretation. This organization is intended to reduce ambiguity in cross-study comparison by separating three questions that are often conflated in the literature: what linguistic unit is decoded, how constrained the candidate output space is, and how the decoded content is ultimately represented to the user.

## 3. Task-Oriented Categorization Framework for Imagined Speech Decoding

The imagined speech literature is highly heterogeneous in task terminology, experimental design, and system objectives. Although many studies can be broadly categorized as imagined speech decoding, they target markedly different linguistic units, ranging from abstract semantic meaning or communicative intent [25,26] to lower-level linguistic units such as vowels, phonemes, syllables [27,28,29,30,31], and from complete words [32,33,34,35,36] to more recent work that has begun exploring the recovery of short sentences, text content, or even speech output [37,38,39,40,41]. Without a consistent task-oriented organizing principle, these studies are easily juxtaposed in ways that obscure differences in task difficulty, methodological suitability, and practical communicative value [16].

**Figure 3:**
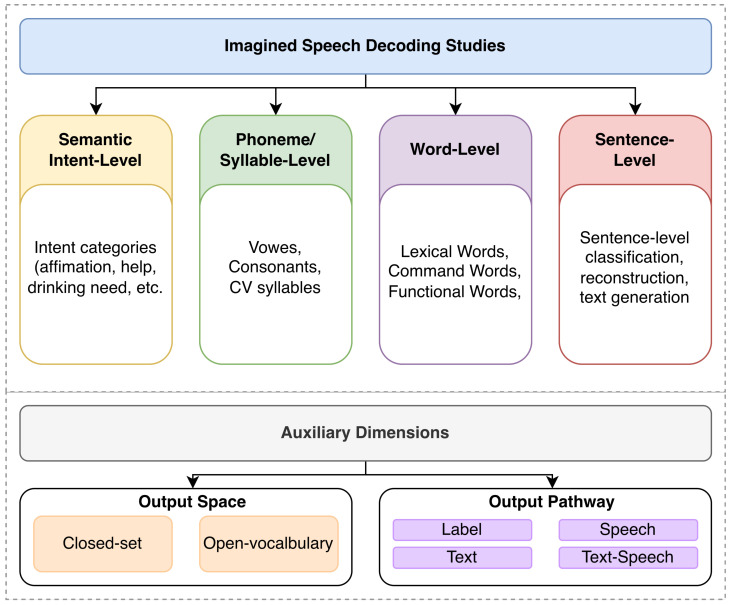
Overview of the task-oriented framework proposed in this review. Imagined speech decoding studies are organized into four main task levels and further characterized by two auxiliary dimensions, namely output space and output pathway.

To address this issue, the present review adopts a task-oriented categorization framework, illustrated in Figure 3. The primary organizing axis is the linguistic level of the unit that the model attempts to recover from neural signals. Based on this principle, the literature is organized into four main task levels, namely semantic or intent-level, phoneme or syllable-level, word-level, and sentence or language-level decoding. This main axis is intended to answer the question of what is being decoded.

To reduce ambiguity in assigning studies to these levels, classification was based on the main decoding objective reported by the original study rather than only on the surface form of the output label. For example, functional items such as “yes,” “no,” or “help” were treated as semantic/intent-level targets when the study defined them primarily as communicative intentions or command states, but as word-level targets when they were evaluated as lexical items within a fixed vocabulary. Similarly, fixed phrases were assigned to sentence/language-level decoding when phrase or sentence structure was central to the task, but were treated as closed-set command labels when used only as functional control categories. Phonemes and syllables were grouped as low-level phonological targets because both are below the lexical word level and are commonly evaluated as constrained unit-classification tasks, although their linguistic differences are acknowledged in the corresponding subsection.

On top of this main hierarchy, two auxiliary dimensions are introduced to improve cross-study comparability. The first is the output-space property, which distinguishes whether a task operates in a closed-set setting or in an open-vocabulary or open-ended setting [37,38,40]. The second is the output pathway, which refers to whether the decoded result is finally expressed as a discrete label, text, or speech, corresponding to neural-signal-to-label, neural-signal-to-text, neural-signal-to-speech, and cascaded neural-signal-to-text-to-speech systems [32,39,41,42,43]. These two auxiliary dimensions do not replace the four main task levels, but further characterize how studies within the same task level are implemented and expressed.

To make the proposed taxonomy more operational, Table 2 provides representative examples showing how the classification criteria can be applied to concrete studies. These examples cover different linguistic target levels and output pathways, ranging from syllable-level closed-set classification to word/fixed-phrase functional decoding and constrained sentence-level text generation.

### 3.1. Semantic or Intent-Level Decoding

Semantic or intent-level tasks focus on the communicative meaning, need category, or interactional intent that an individual wishes to express, rather than the specific lexical or phonological form internally generated in the mind. Representative examples include decoding of semantic categories from silent speech imagination tasks [26,44] and low-bandwidth communication settings based on affirmative or negative responses [45,46]. In such tasks, the model is intended to recover an abstract semantic or communicative category rather than only a specific lexical item.

This level is particularly meaningful in assistive communication scenarios because it may support relatively stable expression at low information rates. For users with severe motor and speech impairments, a system does not necessarily need to recover full sentences to be practically useful. Reliable recognition of basic communicative intents such as agreement, rejection, or basic need-related categories may already provide substantial functional benefit. For this reason, semantic or intent-level decoding can be viewed as a practical transitional route toward usable imagined speech communication systems.

It should be noted that the boundary between semantic/intent-level tasks and word-level tasks is not absolute, because words themselves often carry meaning. Terms such as *yes*, *no*, *help*, and *water* are both lexical items and communicative signals. To improve consistency in literature organization, the present review does not classify studies based solely on whether a word carries meaning. Instead, the distinction is made primarily according to the level of the supervision labels. Specifically, a task is categorized as semantic or intent-level if the model distinguishes abstract needs, intentions, or semantic categories, and if multiple expressions may map to the same label. Conversely, a task remains word-level if the model distinguishes specific lexical items, even when those words have clear communicative functions.

Representative studies in this category commonly focus on semantic categories, binary communicative responses, or low-bandwidth assistive communication signals [26,44,45,46]. Their primary emphasis is usually communicative usability rather than high-expressiveness language recovery. Accordingly, this category should not be directly compared with higher-vocabulary lexical tasks or sentence-level reconstruction studies without careful qualification.

### 3.2. Phoneme or Syllable-Level Decoding

Phoneme or syllable-level tasks target relatively small linguistic units, including vowels [27,28,33,47,48,49,50,51,52,53,54,55,56,57,58], consonants [59,60,61,62], phoneme classes [60,63,64,65], syllable units [29,50,54,66,67], and suprasegmental features such as tone [31,68,69]. These studies usually address a more fundamental question: whether neural signals recorded without overt articulation contain decodable information sufficient to distinguish lower-level speech units. Compared with semantic or lexical tasks, phoneme and syllable tasks often involve more tightly controlled experimental settings and smaller output spaces, which makes them particularly useful as testbeds for investigating the basic discriminability of imagined speech. The exact form of such sublexical tasks may also vary across languages. For example, some studies in tonal languages explicitly involve tonal contrasts [68], whereas others focus more on vowels, consonants, phoneme classes, or CV syllables [70,71,72] depending on the linguistic background and experimental design.

Common experimental designs in this category include vowel recognition, such as /a/, /i/, and /u/, etc. [30,33,49,50,51,57,59,60,73,74], consonant or phoneme class classification [54], and CV syllable discrimination [30,73,75]. The theoretical significance of such tasks lies in the fact that they correspond more directly to the basic building blocks of linguistic form, thereby providing a useful way to examine whether imagined speech retains phonological representations comparable to some aspects of overt speech. From a practical communication perspective, however, these tasks are typically insufficient on their own because even if a small number of phonemes can be distinguished reliably, they must still be combined into words or larger units, which greatly increases system complexity.

Although phoneme-level and syllable-level tasks are grouped together in this review, they are not fully equivalent. Phonemes represent the minimal contrastive units of sound, whereas syllables occupy an intermediate position between phonemes and words. Given that the imagined speech literature is still limited in scale and that these task types are often discussed together in methodological comparisons, the present review treats them as a single main category while still distinguishing, where necessary, among vowels, consonants, phoneme classes, syllables, and tonal syllables.

Representative studies at this level are most often conducted in closed-set settings with limited phonological inventories. Their main contribution lies in demonstrating discriminability and in providing evidence for the existence of lower-level speech-related representations, rather than directly supporting high-bandwidth communicative interaction.

### 3.3. Word-Level Decoding

Word-level tasks are among the most common and representative categories in the imagined speech literature. These studies use complete words as the basic decoding unit, achieving a balance among task difficulty, semantic expressiveness, and experimental controllability. Compared with phonemes and syllables, words carry more explicit meaning. Compared with sentences or unconstrained language generation, they are also more amenable to closed-set experimental designs, making relatively stable decoding results easier to achieve.

Within this category, several common subtypes can be distinguished. The first consists of general lexical word tasks, in which ordinary lexical items are selected as imagined speech targets and the main objective is to examine discriminability among specific word forms [32,55,56,76,77,78,79,80]. The second consists of command-word tasks, such as left, right, up, and down, or their equivalents in other languages [33,57,58,74,81,82,83,84,85,86,87,88,89,90]. These words are primarily intended to drive system control or directional operation. In some application-oriented settings, however, the target vocabulary is not limited to pure command words, but may also include mixed task-related lexical items, such as action commands, object terms, and location terms designed for human–machine interaction [91,92,93] or assembly-oriented communication [55,94,95,96]. The third consists of functional communication word tasks, such as *yes*, *no*, *water*, *food*, *sleep*, *help*, or *medicine*, which are typically selected for assistive communication scenarios involving high-frequency need expression [34,35,36,80,97,98,99,100,101,102,103,104,105,106,107]. Some word-level imagined speech studies also use character- or symbol-oriented targets, such as letters, digits, punctuation marks, or Chinese characters [91,108,109,110]. Although these targets are not conventional lexical words, they are still more appropriately treated as word-level closed-set symbolic classification when the model is trained to discriminate complete named targets rather than sublexical speech units [66,67,111]. Some imagined speech studies use short fixed phrases rather than isolated single words. Even when the targets take the form of multi-word expressions, they are still more appropriately treated as word-level closed-set tasks when the model is trained to discriminate a small set of fixed command- or communication-oriented phrases [42,112,113].

It is important to note that although functional communication word tasks are closely related to semantic or intent-level applications, their supervision targets still involve discrimination among specific lexical items. For this reason, their main classification should remain at the word level rather than being elevated to the semantic level. In the present review, such tasks are described through a combination of main categories and modifying attributes. For example, studies may be characterized as word-level closed-set decoding with functional communication vocabulary, which preserves both its linguistic-unit identity and its communicative application context [32,114,115,116].

From an application perspective, word-level tasks occupy an important intermediate position between lower-level linguistic forms and higher-level communicative expression. On the one hand, they preserve meaningful linguistic content; on the other, they avoid the extreme data and modeling burdens associated with sentence-level tasks. As a result, word-level decoding constitutes the dominant experimental setting in much of the EEG imagined speech literature and often serves as the basis for both command-oriented interaction and assistive communication expansion.

### 3.4. Sentence or Language-Level Decoding

Sentence- or language-level tasks target linguistic content beyond isolated words. The target may take the form of short phrases, full sentences, text sequences, or more open-ended language expressions. This category is often regarded as an important step toward naturalistic communication because it no longer aims only to recover isolated lexical items, but instead seeks to output language segments that are closer to realistic human expression.

The research forms within this category vary substantially. Some studies are classified into a fixed set of predefined sentences. Others attempt to reconstruct textual content, including continuous semantic reconstruction from non-invasive brain recordings [38,117,118] and more open-vocabulary neural communication settings [37,118,119], synthesize sentence-level speech from neural signals [41,43,120], or generate dynamic speech-related outputs such as viseme-based visual speech reconstruction [121]. Still others incorporate pretrained language models and align neural representations with textual embedding spaces to support more complex neural-signal-to-text or neural-signal-to-speech systems [37,40,122,123,124]. However, strictly defined imagined speech studies that directly integrate LLMs for high-level language generation remain limited. Most existing LLM-related work has instead been developed in broader neural-signal-to-text settings or in brain-to-language paradigms that do not strictly correspond to imagined speech [122,123,125]. A key point here is that sentence-level tasks do not automatically imply open-vocabulary generation [39]. If the system simply selects one label from a fixed sentence set, then the task remains a closed-set classification problem. Only when the system attempts to recover previously unseen sentences, reconstruct language in a more open lexical space, or explicitly target unconstrained speech does it become more appropriate to classify the task as open-vocabulary.

From a research perspective, sentence or language-level tasks are the closest to the ultimate goal of natural brain–computer communication, but they also face the greatest methodological difficulty. While these tasks impose stronger demands on data scale, annotation quality, temporal alignment, and model capacity, the introduction of language models and generative approaches makes higher-level outputs increasingly susceptible to language priors, thus creating potential ambiguity between apparently fluent generation and actual neural contribution [38,40,122,123]. For this reason, sentence or language-level tasks are both among the most attractive future directions in imagined speech research and among the most in need of careful task definition, output-space distinction, and explicit control analysis.

## 4. Cross-Cutting Auxiliary Dimensions and Cross-Category Analysis

### 4.1. Output-Space Property

The output-space property describes whether the candidate output space of a task is predefined or open. If the model must choose among a fixed set of candidate labels, the task is categorized as closed-set decoding. If the system is allowed to produce text or speech content beyond a fixed lexicon or sentence set, the task is categorized as open-vocabulary or open-ended decoding [37,38,39,40,123]. This dimension is related to task level, but the two are not equivalent.

Most early imagined speech studies fall into closed-set settings, including vowel classification, syllable recognition, small-vocabulary command-word decoding, and fixed-sentence classification [27,28,30,33,34,35,112]. Such tasks benefit from strong experimental control, clear evaluation metrics, and better suitability for small-sample studies investigating whether decodable information is present in neural signals. However, high accuracy in closed-set settings should not be interpreted automatically as evidence of strong natural-language communication ability, because the output space is already heavily constrained by experimental design.

Open-vocabulary settings are closer to natural communication, especially in sentence-level text reconstruction and unconstrained speech recovery studies [37,38,40,122,123]. However, they also impose much stronger demands on data scale, model robustness, and evaluation design. A key methodological issue is that sentence-level reconstruction systems may appear more expressive while at the same time relying more strongly on linguistic priors [38,40,122,123]. Therefore, any discussion of open-vocabulary imagined speech should consider not only output fluency, but also whether the recovered content can be meaningfully attributed to neural input.

A particularly important clarification is that sentence-level tasks do not automatically imply open-vocabulary settings. If a system operates over a fixed set of sentence labels, then it remains methodologically closer to closed-set classification even though the labels themselves are full sentences [39]. Conversely, a study that attempts to reconstruct previously unseen textual or speech content should be regarded as open-vocabulary even when the final linguistic unit is relatively short [37,38,40].

### 4.2. Output Pathway

Output pathway refers to the form in which imagined speech decoding results are finally expressed. In this review, the existing systems are grouped into three major direct pathways and one cascaded pathway. The first is neural-signal-to-label, where the system outputs a discrete class label. Such labels may correspond to intent categories, phoneme classes, word identities, or fixed sentence classes. The second is neural-signal-to-text, where the system directly outputs textual content such as words, phrases, or sentence strings. The third is neural-signal-to-speech, where the system directly outputs acoustic representations, including mel spectrograms, mel-frequency cepstral coefficients (MFCCs), spectral features, or speech waveforms. The fourth is neural-signal→text→speech, where the system first recovers labels or text from neural signals and then converts them into audible speech through a text-to-speech module [39,41,43,123,126].

Neural-signal-to-label remains the most common pathway in existing imagined speech studies, especially in closed-set semantic, phonological, and word-level tasks [25,26,27,28,30,32,33,34,35]. Its main advantage lies in methodological simplicity and relatively clear evaluation criteria. However, this pathway is also the most limited in expressive capacity, since it reduces communication to selecting among a predefined set of labels.

Neural-signal-to-text represents a more direct route toward symbolic language recovery. Compared with label-based decoding, this pathway is more naturally aligned with language-level expression and can potentially support more flexible interaction [37,38,39,40,117,118,122,123]. At the same time, it also introduces stronger dependence on textual priors and makes it more difficult to disentangle genuine neural contribution from language-model assistance [38,40,122,123].

Neural-signal-to-speech emphasizes direct acoustic reconstruction rather than symbolic text output. This pathway is particularly attractive from the perspective of natural communication because it aims to generate audible speech directly from neural signals. However, it also faces substantial technical difficulty, including the need to reconstruct meaningful acoustic detail from weak and noisy biosignals [41,43,120,126]. In some recent work, this notion has also been extended to dynamic speech-related outputs, such as viseme-based visual speech reconstruction, which broadens the meaning of speech-related communication beyond conventional acoustic synthesis [121].

The cascaded neural-signal→text→speech pathway occupies an intermediate position. In this setting, neural signals are first decoded into labels or text, and a subsequent text-to-speech system is used to generate speech. Although this pathway is not equivalent to direct neural-signal-to-speech reconstruction, it may be more realistic in communication-oriented applications because it allows textual verification, error correction, and user-in-the-loop interaction before speech synthesis [39,123].

A critical distinction must be maintained between the output pathway and the task level. The output pathway describes how the decoded content is represented and delivered, whereas the task level describes what linguistic unit the system aims to recover. These two dimensions are related, but they should not be conflated. For example, a sentence-level task may still be implemented as neural-signal-to-label if it selects among fixed sentence categories, while a word-level task may in principle adopt neural-signal-to-text or neural-signal-to-speech outputs [39,40,41,42].

The output pathway also affects how results should be evaluated. Neural-signal-to-label systems are usually interpreted through classification-oriented metrics, such as accuracy or F1-score, whereas neural-signal-to-text and neural-signal-to-speech systems require sequence-, semantic-, acoustic-, or perceptual-level evaluation depending on the output form. In cascaded neural-signal→text→speech systems, errors from the neural-to-text stage may further propagate to or be smoothed by the downstream speech-generation module. For generative text or speech pathways, evaluation should also consider whether the output is genuinely constrained by neural signals or partly driven by language priors and downstream generative modules. Therefore, the output pathway should be reported together with the task level and output-space property when comparing imagined speech decoding studies.

### 4.3. Interactions Between Main Task Levels and Auxiliary Dimensions

The auxiliary dimensions discussed above do not operate independently of the main task hierarchy. Instead, the current literature suggests several recurring patterns. To provide a more explicit overview of these cross-dimensional patterns, Table 3 summarizes the distribution of the 103 core original studies across the four task levels and the main empirical output pathways.

As shown in Table 3, the current literature remains heavily concentrated in neural-signal-to-label studies, especially at the phoneme/syllable and word levels. Semantic-, phoneme-, syllable-, and most word-level studies are predominantly conducted in closed-set settings and commonly rely on neural-signal-to-label outputs [25,26,27,28,30,32,33,34,35]. This reflects both the practical difficulty of imagined speech decoding and the historical tendency to prioritize discriminability verification over expressive communication. The distribution also shows that more expressive output pathways remain comparatively underexplored.

By contrast, sentence- or language-level studies are more likely to move toward open-vocabulary settings and toward neural-signal-to-text or neural-signal-to-speech pathways [37,38,39,40,41,122,123]. These directions are more closely aligned with the long-term goal of naturalistic brain–computer communication, but they also involve greater methodological uncertainty. In particular, as studies become more expressive in output form, they also become more vulnerable to confounding by linguistic priors, multimodal auxiliary signals, and post-processing stages [38,40,122,123].

In practical communication systems, the most realistic pathway may vary depending on the system’s objectives. If the goal is reliable low-bandwidth communication, closed-set neural-signal-to-label systems may remain the most feasible option. If the goal is richer, more natural communication, neural-signal-to-text and cascaded neural-signal→text→speech systems may offer a more practical intermediate route than direct neural-signal-to-speech. Direct speech reconstruction remains highly attractive, but its current technical burden is substantially higher [39,41,43,123].

Overall, cross-category analysis shows that task level, output-space property, and output pathway jointly determine how an imagined speech study should be interpreted and compared. A high-accuracy closed-set word classification task and a lower-scoring open-vocabulary text reconstruction task may reflect fundamentally different task difficulty and communicative ambition. Meaningful comparison, therefore, requires explicit reporting of these dimensions rather than relying solely on headline performance metrics. In addition, this framework helps clarify boundary cases that are often conflated in existing discussions, such as semantic intent versus lexical decoding, sentence-level classification versus open-vocabulary generation, and output pathways versus primary task categories.

## 5. Discussion

### 5.1. Methodological Evolution of Imagined Speech Decoding

The development of imagined speech decoding research shows a clear methodological progression from feasibility-oriented classification toward more expressive and communication-oriented neural language processing [2,4,5,10,12,14]. Early studies were mainly concerned with whether imagined speech contains discriminable neural information at all. For this reason, much of the initial literature focused on small closed-set tasks, low-level linguistic units, and conventional machine-learning pipelines built on handcrafted features and shallow classifiers [2,4,27,28,32,59,90]. In this stage, the main objective was not yet to support rich communication, but rather to verify that imagined speech-related neural activity could be separated from other mental states or among a small number of target classes under controlled conditions [2,4,11].

As the field progressed, deep-learning-based approaches became increasingly dominant. Convolutional, recurrent, attention-based, and transformer-inspired architectures enabled more flexible modeling of temporal, spatial, and cross-channel structure in neural signals [28,29,32,35,52,98,111,127]. These methods often improved performance in word-level and phoneme-/syllable-level tasks, especially when compared with earlier handcrafted feature pipelines [28,32,52,97,106,111]. At the same time, however, the main experimental setting in much of the literature remained relatively conservative, namely small-sample, within-subject, and closed-set classification [5,10,11,12]. In this sense, methodological sophistication increased faster than task realism. Many studies achieved better decoding performance, but the practical communication implications of such gains remained limited by restricted vocabularies and tightly controlled experimental conditions [5,12,14].

A further stage of development is marked by the growing use of multimodal and cross-domain strategies. Representative public datasets and related multimodal or invasive resources included in this review are summarized in Table 4. Additional dataset-level diagnostic details, including channels, sampling rate, trial/session structure, preprocessing notes, evaluation settings, and access-related limitations, are provided in Appendix Table A4. As shown in Table 4, publicly reusable resources in this research area remain predominantly EEG-based, with relatively limited standardized resources in other modalities. In addition to EEG alone, recent studies have explored combinations with EMG, speech-related biosignals, or auxiliary representation spaces [68,77,120]. This trend reflects an important shift in emphasis. Instead of treating imagined speech decoding as a purely single-modality pattern-recognition problem, newer work increasingly attempts to stabilize or enrich weak neural evidence through complementary information sources, cross-modal alignment, or intermediate reconstruction objectives [77,120,126]. Such strategies can improve performance and may provide more robust pathways toward practical systems. However, they also complicate interpretation, because the relative contribution of neural input and auxiliary information becomes harder to disentangle [10,120,126].

More recently, the field has begun shifting from low-level closed-set decoding toward higher-level language recovery. Sentence-level reconstruction, text generation, speech synthesis, viseme-based dynamic output, and LLM-assisted neural language generation all indicate that the research frontier is gradually moving from discriminability verification toward expressive communication [37,38,39,40,41,43,121,122,123]. This shift is conceptually important. It suggests that imagined speech research is no longer confined to determining whether a small set of classes can be distinguished, but is increasingly concerned with how language-like outputs can be generated from non-overt neural activity [37,38,40,122]. Nevertheless, this transition remains uneven. In particular, strictly defined imagined speech studies that directly integrate LLMs for high-level language generation are still relatively limited, and many recent LLM-related advances are found instead in broader neural-signal-to-text or brain-to-language paradigms rather than in narrowly defined imagined speech settings [122,123,125].

Taken together, these developments suggest that imagined speech decoding is evolving along two interacting axes. One axis concerns model complexity, progressing from conventional classifiers to deep neural networks, multimodal fusion, and generative frameworks [4,12,14,111,122,123]. The other concerns output ambition, progressing from low-level closed-set classification to higher-level text and speech-related generation [37,38,39,40,41,43]. The most important implication is that methodological progress should not be measured solely by improved accuracy or by the apparent fluency of generated outputs. Rather, it should be evaluated in relation to the type of task being solved, the structure of the output space, the transparency of the output pathway, and the extent to which the final system actually advances practical brain–computer communication [5,10,12,14].

### 5.2. Challenges and Future Directions

**Figure 4:**
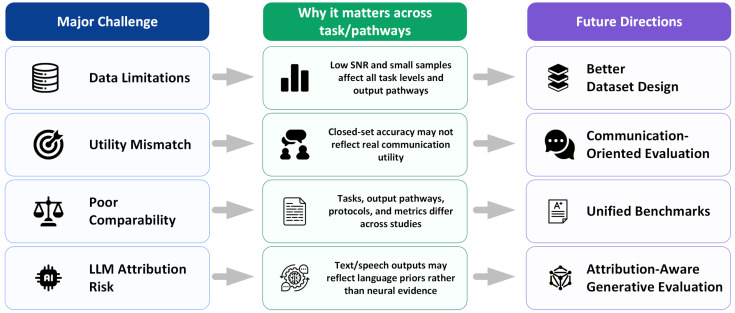
Overview of the major challenges and future directions in imagined speech decoding from a task- and pathway-aware perspective. The figure summarizes how unresolved issues such as data scarcity, subject variability, evaluation uncertainty, language-prior contamination, and practical communication utility differ across closed-set label decoding, neural-signal-to-text generation, neural-signal-to-speech reconstruction, and open-vocabulary or LLM-assisted settings.

Despite rapid methodological progress, several major challenges remain unresolved. Although many of these challenges have been discussed in previous BCI studies, their implications differ across task levels, output-space properties, and output pathways. Therefore, they should be interpreted in relation to the specific decoding target, output constraint, and output form of each study, rather than treated as uniform obstacles across all imagined speech decoding systems. Figure 4 provides an overview of the main challenge–direction relationships discussed in this subsection. A first challenge is that advances in model architecture do not eliminate the fundamental limitations of imagined speech data. Neural signals remain weak, noisy, temporally uncertain, and strongly subject-dependent, especially in non-invasive settings [2,3,4,10,11,12,13]. This limitation also affects where different recording modalities may realistically fit within the task hierarchy. EEG remains practical and scalable, but its low signal-to-noise ratio and limited spatial resolution make sentence-level, open-vocabulary, or generative decoding particularly challenging [2,4,5,12]. In contrast, ECoG and sEEG provide higher spatial specificity and may better support higher-level language reconstruction or neural-signal-to-speech pathways, although their invasiveness limits broad deployment [41,43,143]. As tasks move from phoneme- or word-level classification toward sentence-level reconstruction or generative output, these difficulties become more severe rather than less [37,38,40,122,123]. Larger models may fit richer structure, but they also require stronger supervision, better temporal alignment, and greater data scale than most current imagined speech datasets can provide [5,12,14].

A second challenge concerns the growing gap between decoding performance and communicative usefulness. In closed-set settings, especially those involving small phonological or lexical inventories, relatively high accuracy may be achievable under controlled conditions [27,28,32,34,35]. However, such performance does not necessarily imply that the system can support natural or flexible communication [5,12,14]. Conversely, higher-level systems that generate text or speech are often more attractive from an application perspective, but they are also more difficult to evaluate rigorously [37,38,40,41,43]. This is particularly true when generated outputs may reflect not only neural evidence, but also language priors, multimodal auxiliary signals, or downstream generation modules [38,122,123]. As a result, future studies should be careful not to equate more fluent output with stronger neural decoding without explicit evidence [38,122,123].

A third challenge lies in comparability across studies. The current literature varies substantially in linguistic target level, output-space property, output pathway, evaluation protocol, subject split, dataset scale, and language background [4,5,10,12,14]. For this reason, comparisons based only on headline metrics are often misleading. A word-level closed-set classification task should not be directly compared with an open-vocabulary sentence reconstruction setting, just as direct neural-signal-to-speech synthesis should not be evaluated in exactly the same way as neural-signal-to-label prediction [37,38,41,43]. More explicit reporting of task level, label structure, output pathway, and evaluation setting would, therefore, improve the interpretability of future work [5,12,14,143].

To provide a more concrete comparison under shared datasets and comparable task settings, Table 5 summarizes representative reported results from studies using the same public datasets and closely matched target subsets. Because imagined speech studies often differ in preprocessing, sample construction, validation protocols, and subject averaging, these results should be interpreted as representative within-dataset comparisons rather than unified benchmark rankings.

As shown in Table 5, even when studies use the same public dataset and similar target subsets, reported performance can vary substantially. For example, the two KaraOne studies both address four-word imagined speech classification, but their reported accuracies differ markedly, likely reflecting differences in feature construction, preprocessing, sample generation, and validation strategies. The ASU comparisons provide more closely matched task subsets across studies, including long words, short words, vowels, and short-long words. These examples reinforce the need to report the dataset, target subset, output space, and evaluation protocol explicitly before interpreting performance differences across imagined speech studies.

Language and dataset design also deserve greater attention. The linguistic units used in imagined speech experiments are not fully equivalent across languages. Tonal contrasts, syllabic structures, character-based targets, and language-specific command vocabularies all affect task design and difficulty [31,33,68,92,114,132]. In addition, several public datasets span more than one task level, which means that datasets should not be treated as if they inherently belong to a single category [128,129,130]. Future benchmark design would benefit from clearer annotation of linguistic unit type, output-space property, and intended communication function [131,132,133,144].

The emergence of LLMs and other generative models opens a promising but methodologically delicate direction. These models may help bridge weak neural signals and rich language outputs, especially in sentence-level or text-generation settings [122,123]. However, the central question is no longer only whether the final output appears meaningful, but whether it can be meaningfully attributed to the neural input [38,122,123]. This issue is likely to become one of the defining methodological questions of the next stage of imagined speech research. Stronger ablation studies, neural-only baselines, unseen-content evaluations, and more explicit attribution analyses will be necessary if LLM-assisted decoding is to become a credible imagined speech paradigm rather than merely a fluent post-processing layer [37,38,122,123]. Specifically, attribution-aware generative evaluation should include prompt-only baselines, random or temporally shuffled neural-signal baselines, label-permutation controls, neural-only versus text-only ablations, explicit distinction between zero-shot, few-shot, and supervised evaluation settings, and evaluation on unseen words, sentences, sessions, and subjects. These controls can help distinguish genuine neural contribution from language priors, prompt leakage, dataset memorization, and downstream generative smoothing.

From the perspective of practical system design, future progress may not come from a single universal solution. Different communication objectives may favor different regions of the task space. For low-bandwidth but reliable communication, semantic-/intent-level or small closed-set word-level systems may remain the most feasible [26,34,35,44,45,46,144]. For more flexible symbolic communication, neural-signal-to-text systems may provide a realistic intermediate route, especially when textual verification and correction are possible [37,39,123]. Direct neural-signal-to-speech reconstruction remains highly attractive as a long-term goal, but its technical burden is currently higher and its interpretability often weaker [41,43]. In this sense, the most useful future systems may not be the most ambitious in output form, but the ones that best balance expressiveness, robustness, transparency, and real communicative utility [143].

The present review also has limitations. The proposed task-oriented framework is intended as a practical organizing structure rather than a rigid universal taxonomy. Some studies lie near the boundaries between categories, especially those involving communicative words, fixed phrases, mixed symbolic targets, or multimodal generative pipelines [34,42,66,120,123]. In addition, some recent LLM-related or brain-to-language studies are highly relevant to future imagined speech research, even when they do not strictly satisfy a narrow imagined speech definition [122,123]. These ambiguities do not invalidate the framework, but they do indicate that future refinement may be needed as the field develops.

Overall, the field appears to be moving from proof-of-concept classification toward richer neural communication systems [5,10,12,14,37,38,122,123]. The central challenge for the next stage is not only to decode more, but to decode more meaningfully. Future progress should therefore not be judged only by higher accuracy or more fluent output, but also by task definition, cross-study comparability, interpretability, robustness, and practical communicative usefulness. Continued advances in dataset design, evaluation protocols, multimodal modeling, and high-level generative methods should be accompanied by clearer reporting of what is being decoded, how it is expressed, and what communication goal the system is intended to serve.

Based on the task-oriented analysis in this review, future imagined speech studies should explicitly report a minimal set of comparable information, including the linguistic target level, output-space property, output pathway, dataset and target subset, subject/session split, leakage-control strategy, pathway-appropriate metrics, and attribution controls for generative or LLM-assisted systems. Such reporting would make future studies more comparable, reproducible, and practically interpretable.

## 6. Conclusions

This review has examined imagined speech decoding from a task-oriented perspective. Rather than treating imagined speech as a single homogeneous problem, it argues that the literature is better understood as a family of related but distinct tasks that differ in linguistic target level, output-space property, and output pathway. Based on this view, existing studies were organized into four main task levels, namely semantic-/intent-level, phoneme-/syllable-level, word-level, and sentence-/language-level decoding, and were further interpreted through two auxiliary dimensions: closed-set versus open-vocabulary output space, and neural-signal-to-label, neural-signal-to-text, neural-signal-to-speech, and cascaded neural-signal→text→speech output pathways.

From this analysis, several conclusions can be drawn. First, the imagined speech literature is substantially more heterogeneous than is often assumed, and many reported results are not directly comparable unless task level, output constraint, and system objective are explicitly taken into account. Second, much of the current evidence remains concentrated in closed-set, low- to mid-level decoding tasks, especially phonological and word-level settings, where methodological control is stronger but communicative expressiveness is limited. Third, higher-level directions, including text reconstruction, speech synthesis, and LLM-assisted neural language generation, are beginning to expand the scope of the field, but they also introduce greater methodological uncertainty, particularly in separating genuine neural contribution from linguistic priors and downstream generative effects.

Overall, imagined speech research appears to be moving from proof-of-concept classification toward richer neural communication systems. In this sense, the task-oriented framework proposed in this review is not only a way of organizing past work, but also a guide for interpreting future developments in imagined speech brain–computer communication.

## Figures and Tables

**Table 1:** Representative existing reviews related to imagined speech decoding and closely related speech-BCI topics.

Review	Main Focus	Main Organization	Task-Level Taxonomy	Output-Space Distinction	Output-Pathway Analysis
Panachakel and Ramakrishnan, 2021 [2]	EEG-based covert and imagined speech decoding methods	Pipeline/method-oriented	Partial	No	Limited
Rahman et al., 2024 [3]	EEG speech imagery decoding for BCI communication	Method/progress-oriented systematic review	Partial	Limited	Limited
Lopez-Bernal et al., 2022 [4]	EEG-based imagined speech datasets, features, and classifiers	Dataset/feature/classifier-oriented	Partial	No	No
Alzahrani et al., 2024 [11]	EEG-based imagined speech classification	Classification-method-oriented	Partial	No	No
Tates et al., 2025 [5]	Speech imagery BCI methods and real-time progress	Systematic literature review	Partial	Limited	Limited
Su and Tian, 2025 [10]	EEG-based speech imagery decoding and encoding	Progress-oriented systematic review	Partial	Limited	Limited
Jin et al., 2025 [12]	EEG-based imagined speech decoding over the last decade	Theory/data/feature/model-oriented	Partial	Limited	Limited
Fitriah et al., 2022 [13]	Silent speech interfaces for assistive communication	Challenge/system-oriented	Limited	No	Limited
Zhang et al., 2025 [14]	Deep learning for EEG speech imagery decoding	Deep-learning-method-oriented survey	Partial	No	No
Shah et al., 2022 [17]	AI methods for EEG-based speech decoding	AI-method-oriented scoping review	Partial	No	Limited
Gonzalez-Lopez et al., 2020 [18]	Silent speech interfaces for speech restoration	Application/restoration-oriented	Limited	No	Limited
Cooney et al., 2022 [19]	Experimental protocols for speech-related neural studies	Protocol/design-oriented	Limited	No	No
Tang et al., 2024 [20]	Imagined speech reconstruction from neural signals	Source/reconstruction-oriented overview	Partial	Limited	Partial
Almufareh et al., 2025 [21]	Inner speech decoding from neural signals	Inner-speech-oriented review	Partial	Limited	Limited
Shrividya et al., 2025 [22]	Non-invasive imagined speech decoding and fluency gap	Technique/challenge-oriented	Partial	No	Limited
Present review	Task-oriented imagined speech decoding in BCI	Task-oriented framework	Yes	Yes	Yes

*Note:* “Yes” indicates that the dimension is used as a primary organizing axis; “Partial” indicates that the topic is discussed but not used as the main framework; “Limited” indicates brief or indirect coverage; and “No” indicates that the dimension is not systematically analyzed.

**Table 2:** Representative examples for applying the proposed taxonomy.

Study	Task Target	Task Level	Output Space	Output Pathway	Taxonomic Interpretation
Wu et al. [30]	Two imagined syllables for real-time BCI control	Phoneme/syllable-level	Closed-set	Neural-signal-to-label	Demonstrates discrimination of predefined sublexical speech units; should not be interpreted as word-, semantic-, or sentence-level decoding.
Fitriah et al. [34]	Predefined communicative words and short expressions, e.g., “yes,” “no,” “stop,” “help me,” and “thank you”	Word-level/fixed-phrase functional communication	Closed-set	Neural-signal-to-label	Evaluates discrimination among fixed communicative labels rather than open-ended language generation.
Pan et al. [39]	Twenty imagined short sentences represented through character-level labels	Sentence/language-level	Constrained text generation	Neural-signal-to-text	Moves beyond label classification by generating text-like output, but remains different from fully open-vocabulary language generation.

**Table 3:** Distribution of the core original studies across task levels and output pathways.

Task Level	Neural-Signal-to-Label	Neural-Signal-to-Text	Neural-Signal-to-Speech/Speech-Related Output	Total
Semantic/intent-level	5	0	0	5
Phoneme/syllable-level	26	0	0	26
Word-level	54	1	1	56
Sentence/language-level	1	10	5	16
Total	86	11	6	103

*Note:* Each study was assigned to its dominant task level and dominant output pathway according to the primary decoding objective reported in the original paper. Studies involving mixed targets were counted once based on their dominant task objective. Fixed words, phrases, or sentences were counted as neural-signal-to-label tasks when the system selected among predefined candidates rather than generating unconstrained text or speech.

**Table 4:** Representative public datasets and related multimodal or invasive resources included in this review.

Dataset/Resource	Modality	Subjects	Primary Target	Task Level	Task Subtype/Notes
KaraOne [128]	EEG	12	7 phonemic/syllabic prompts; 4 lexical words	Phoneme-/syllable-level + word-level	Mixed-level dataset
ASU [129]	EEG	15	Vowels; short words; long words	Phoneme-/syllable-level + word-level	Mixed-level dataset
Coretto DB [130]	EEG	15	5 vowels; directional command words	Phoneme-/syllable-level + word-level	Mixed-level dataset
TOL [131]	EEG	10	Direction words	Word-level	Command-word task
Chisco [132]	EEG	5	Semantic-category sentences/phrases	Sentence-/language-level	Large-scale fixed-sentence closed-set corpus
Words6 [133]	EEG	15	Six imagined words	Word-level	General lexical word task
FEIS [134]	EEG	21	16 English phonemes	Phoneme-/syllable-level	Low-channel imagined speech dataset
3M-CPSEED [135]	EEG	20	Chinese pinyin/syllables across overt, mouthed, and imagined speech	Phoneme-/syllable-level	Chinese multi-mode dataset
ArEEG [136]	EEG	12	Five Arabic inner-speech commands	Word-level	Command-word dataset; 8-channel setup
DAIS [137]	EEG + speech	20	15 Dutch prompts	Word-level	Articulated vs. imagined speech comparison dataset
Pragmatic Mandarin multimodal DB [138]	EEG + sEMG + speech	30	Mandarin speech patterns under overt, silent, and imagined modes	Mixed-level	Public multimodal Mandarin resource
Bimodal EEG-fMRI inner-speech DB [139]	EEG + fMRI	4	8 words from social/numerical categories	Word-level + semantic-/intent-level	Nonsimultaneous bimodal dataset
Simultaneous EEG-fMRI inner-speech DB [140]	EEG + fMRI + ECG	3	8 words from social/numerical categories	Word-level + semantic-/intent-level	Simultaneous multimodal dataset
VocalMind [141]	sEEG + speech	1	Mandarin words and sentences	Word-level + sentence-/language-level	Invasive resource; vocalized/mimed/imagined speech
Semantic EEG-fNIRS DB [142]	EEG + fNIRS	12 (+7 EEG-only follow-up)	Semantic categories (animals vs. tools) under silent naming and sensory imagery tasks	Semantic-/intent-level	Related multimodal resource

**Table 5:** Representative reported comparisons under shared datasets and comparable task settings.

Dataset	Task/Target Subset	Study	Metric	Reported Performance
KaraOne	Four-word imagined speech classification: *pat*, *pot*, *knew*, *gnaw*	Bisla and Anand [66]	Accuracy	43.76%
KaraOne	Four-word imagined speech classification: *pat*, *pot*, *knew*, *gnaw*	Zheng et al. [100]	Accuracy	80.51%
ASU	Long words: *independent* vs. *cooperate*	Panachakel and Ganesan [55]	Accuracy	88.82%
ASU	Long words: *independent* vs. *cooperate*	Kamble et al. [56]	Accuracy	94.82%
ASU	Short words: *in*, *out*, *up*	Panachakel and Ganesan [55]	Accuracy	83.95%
ASU	Short words: *in*, *out*, *up*	Kamble et al. [56]	Accuracy	94.68%
ASU	Vowels: /*a*/, /*i*/, /*u*/	Panachakel and Ganesan [55]	Accuracy	86.28%
ASU	Vowels: /*a*/, /*i*/, /*u*/	Kamble et al. [56]	Accuracy	84.50%
ASU	Short-long words: *in* vs. *cooperate*	Panachakel and Ganesan [55]	Accuracy	92.80%
ASU	Short-long words: *in* vs. *cooperate*	Kamble et al. [56]	Accuracy	94.26%

*Note:* Values are reported directly or calculated from subject-wise results in the original papers; they are intended for representative within-dataset comparison rather than unified benchmark ranking.

## Data Availability

Not applicable.

## Abbreviations

The following abbreviations are used in this manuscript:

LLMs	Large Language Models
CNN	Convolutional Neural Network
RNN	Recurrent Neural Network
LSTM	Long Short-Term Memory
MFCC	Mel-Frequency Cepstral Coefficient
CV	Consonant-Vowel
ECoG	Electrocorticography
MEG	Magnetoencephalography
fMRI	Functional Magnetic Resonance Imaging
fNIRS	Functional Near-Infrared Spectroscopy
EMG	Electromyography
sEEG	Stereoelectroencephalography

## Appendix A

**Table A1:** Search string and settings for Web of Science Core Collection.

Item	Description
Database	Web of Science Core Collection
Search field	Topic
Search string	TS=((“imagined speech” OR “inner speech” OR “speech imagery” OR “covert speech” OR “silent speech”) AND (“EEG” OR “electroencephalography” OR “ECoG” OR “sEEG” OR “MEG” OR “fMRI” OR “fNIRS” OR “brain–computer interface” OR “BCI”) AND (decod* OR classif* OR recognit* OR “neural decoding” OR “machine learning” OR “deep learning” OR CNN OR RNN OR LSTM OR transformer OR “transfer learning” OR “domain adaptation”))
Publication window	1 January 2020 to 6 February 2026
Document type/filter	Article; Review Article; Proceedings Paper; Early Access
Language	English
Query date	6 February 2026

*Note:* The asterisk (*) denotes a wildcard used to retrieve word variants.

**Table A2:** Search string and settings for PubMed.

Item	Description
Database	PubMed
Search field	Title/Abstract
Search string	((“imagined speech”[Title/Abstract] OR “inner speech”[Title/Abstract] OR “speech imagery”[Title/Abstract] OR “covert speech”[Title/Abstract] OR “silent speech”[Title/Abstract]) AND (“EEG”[Title/Abstract] OR “electroencephalography”[Title/Abstract] OR “ECoG”[Title/Abstract] OR “sEEG”[Title/Abstract] OR “MEG”[Title/Abstract] OR “fMRI”[Title/Abstract] OR “fNIRS”[Title/Abstract] OR “brain–computer interface”[Title/Abstract] OR “BCI”[Title/Abstract]) AND (decoding[Title/Abstract] OR classification[Title/Abstract] OR recognition[Title/Abstract] OR “neural decoding”[Title/Abstract] OR “machine learning”[Title/Abstract] OR “deep learning”[Title/Abstract] OR CNN[Title/Abstract] OR RNN[Title/Abstract] OR LSTM[Title/Abstract] OR transformer[Title/Abstract] OR “transfer learning”[Title/Abstract] OR “domain adaptation”[Title/Abstract]))
Publication window	1 January 2020 to 6 February 2026
Document type/filter	Journal Article; Review
Language	English
Query date	6 February 2026

**Table A3:** Search string and settings for IEEE Xplore.

Item	Description
Database	IEEE Xplore
Search field	All Metadata
Search string	((“imagined speech” OR “inner speech” OR “speech imagery” OR “covert speech” OR “silent speech”) AND (“EEG” OR “electroencephalography” OR “ECoG” OR “sEEG” OR “MEG” OR “fMRI” OR “fNIRS” OR “brain–computer interface” OR “BCI”) AND (decoding OR classification OR recognition OR “neural decoding” OR “machine learning” OR “deep learning” OR CNN OR RNN OR LSTM OR transformer OR “transfer learning” OR “domain adaptation”))
Publication window	1 January 2020 to 6 February 2026
Document type/filter	Journals; Conferences; Early Access Articles
Language	English
Query date	6 February 2026

*Note:* Google Scholar was used only for supplementary retrieval, citation tracking, and cross-checking, and was not included in the main structured search counts.

## Appendix B. Dataset Diagnostic Details

**Table A4:** Diagnostic details of representative datasets and resources for imagined speech decoding.

Dataset/Resource	Subjects	Channels	Sampling Rate	Trials/Sessions	Preprocessing Notes	Split/Evaluation	Limitations/Access Notes
KaraOne [128]	12 recruited; 8 used	64 EEG + 4 EOG	1 kHz	132 trials; 4 trial states	EEGLAB; ocular removal; 1–50 Hz; Laplacian	LOSO cross-validation	Mixed phoneme/word targets; small sample; public-release status should be verified
ASU [129]	15	64	1000 Hz raw; 256 Hz processed	1–3 sessions/subject; 100 trials per word/sound	8–70 Hz band-pass; 60 Hz notch; EOG artifact removal	10-fold CV; random train/test split	Published dataset; vowels, short words, and long words; task groups differ across subjects
Coretto DB [130]	15	6	1024 Hz	50 trials/word; single session; approx. 3.5 h	2–40 Hz FIR band-pass; artifact marking	RF/SVM baseline analysis	Spanish vowels and command words; low-channel setup limits spatial analysis
TOL [131]	10	128 EEG + 8 EXG	256 Hz	475–570 trials/subject; 3 sessions; 5640 total trials	MNE-based processing scripts; processed and raw data provided	Dataset validation; no fixed universal benchmark split	Inner speech, pronounced speech, and visualized conditions; task execution cannot be directly verified
Chisco [132]	3	125 EEG + 6 external	1 kHz raw; 500 Hz processed	6681 trials/subject; 5 days; 9 blocks/day	PREP; notch; high-pass; Autoreject; ICA	Semantic-category baseline classification	Large subject-specific dataset; only 3 subjects; sentence-level imagined speech
Words6 [133]	15	64	2048 Hz	50 trials/word; 6 imagined words	CAR; 0.01–250 Hz; 48–52 Hz notch; ICA	Classification baseline	Six-word closed-set task; open-access imagined-word dataset
FEIS [134]	21 English; 2 Chinese	14	256 Hz	Phase-specific CSV files for stimuli, thinking, speaking, and resting states	Raw CSV files; processing scripts provided	No fixed benchmark split reported	Open Zenodo dataset; low-channel Emotiv EPOC+ recording
3M-CPSEED [135]	20	32 or 128	500 Hz/ 1000 Hz raw; 500 Hz processed	4 blocks; 1800 validated trials/subject	Downsampling; 50 Hz notch; 4–45 Hz; Autoreject; ICA	Dataset validation; transfer-learning potential	Different devices across subjects; overt, mouthed, and imagined speech modes
ArEEG [136]	12	8	250 Hz	15 sessions/subject; 25 trials/session; 15 s/trial; 4650 trials total	50 Hz notch; 0.5–30 Hz band-pass; scaling to ±50 μV	80/20 train–test split; participant-specific evaluation	OpenNeuro dataset; Arabic 5-command inner speech; low-density EEG; no subject-independent evaluation reported
DAIS [137]	20	64	1024 Hz	20 runs × 15 trials; 5993 trials total	1–70 Hz; 49–51 Hz notch; re-reference; blink marking	Speaker-independent validation	Dutch articulated and covert speech in the same trial; 62 usable EEG channels for most subjects
Pragmatic Mandarin multimodal DB [138]	30	64 EEG; 6 sEMG	1000 Hz raw; 256 Hz processed EEG	3 modality-homogeneous blocks; 10 materials; 50 repetitions/material; ∼48 min/subject	EEGLAB; bad-channel interpolation; average reference; 0–120 Hz; 50 Hz notch; downsampled; baseline correction; ICA	Mode-level validation; no fixed benchmark split reported	Open dataset with EEG, sEMG, and speech; imagined, silent, and overt Mandarin speech; individual material-level decoding not evaluated
Bimodal EEG-fMRI inner-speech DB [139]	4	64 EEG + 6 external	512 Hz	320 EEG trials; 2 fMRI sessions	EEG ICA analysis; fMRI SPM12	Open code/benchmark; subject-dependent use	OpenNeuro; nonsimultaneous EEG-fMRI; small sample
Simultaneous EEG-fMRI inner-speech DB [140]	3	64 EEG + ECG	5 kHz	2 sessions; 8 words; 40 trials/word; 2-s fixation, 2-s task, 12-s rest	AAS correction for MRI gradient and cardiac artifacts; BIDS EEG/fMRI data	No fixed benchmark split reported	OpenNeuro; CC0 data; small sample; incomplete EEG sessions for sub-01/sub-02
VocalMind [141]	1	140 implanted contacts; 110 used after exclusion	1000 Hz raw; 200 Hz processed	20 words × 6 reps/mode; 100 sentences × 2 reps/mode; 3 speech modes; 67.85 min total	30 abnormal contacts removed; CAR; high-gamma 70–150 Hz; low-frequency signal; downsampled to 200 Hz	Six-fold CV baseline for speech decoding	Zenodo dataset; single sEEG participant; Mandarin tonal speech; vocalized/mimed/imagined modes
Semantic EEG-fNIRS DB [142]	12 (+7 EEG-only follow-up)	64 EEG; fNIRS 11/14 ch	EEG 2048 Hz; fNIRS 8.92/7.81 Hz	36 concepts; 5 reps/concept in Dataset 1; 7 reps/concept in Dataset 2	Raw BIDS data; EEG/fNIRS preprocessing examples reported	No fixed benchmark split reported	OpenNeuro; semantic animals/tools task; task-order seed issue in Dataset 1

*Note:* LOSO = leave-one-subject-out; RF = random forest; SVM = support vector machine. This table provides concise dataset-level diagnostic information rather than a full dataset manual.
